# Cytokine Targeting by miRNAs in Autoimmune Diseases

**DOI:** 10.3389/fimmu.2019.00015

**Published:** 2019-01-29

**Authors:** Valentina Salvi, Veronica Gianello, Laura Tiberio, Silvano Sozzani, Daniela Bosisio

**Affiliations:** Department of Molecular and Translational Medicine, University of Brescia, Brescia, Italy

**Keywords:** TNF-α, IL-6, IL-17/IL-23, IFN, SLE, RA, psoriasis, MS

## Abstract

Persistent and excessive cytokine production is a hallmark of autoimmune diseases and may play a role in disease pathogenesis and amplification. Therefore, cytokine neutralization is a useful therapeutic strategy to treat immune-mediated conditions. MicroRNAs (miRNAs) are small non-coding RNA molecules that regulate gene expression in diverse biological processes. Altered miRNA levels are observed in most autoimmune diseases and are recognized to influence autoimmunity through different mechanisms. Here, we review the impact of altered miRNA levels on the expression of cytokines that play a relevant pathogenic role in autoimmunity, namely primary pro-inflammatory cytokines, the IL-17/IL-23 axis, type I interferons and IL-10. Regulation can be either “direct” on the target cytokine, or “indirect,” meaning that one given miRNA post-transcriptionally regulates the expression of a protein that in turn influences the level of the cytokine. In addition, miRNAs associated with extracellular vesicles can regulate cytokine production in neighboring cells, either post-transcriptionally or via the stimulation of innate immune RNA-sensors, such as Toll-like receptors. Because of their tremendous potential as physiological and pathological regulators, miRNAs are in the limelight as promising future biopharmaceuticals. Thus, these studies may lead in the near future to the design and testing of therapeutic miRNAs as next generation drugs to target pathogenic cytokines in autoimmunity.

## Introduction

Autoimmune diseases are chronic and often life threatening conditions characterized by an undesired activation of the immune system against self-antigens, whose incidence and prevalence has markedly increased over the second half of the twentieth century (1). The pathogenesis of these diseases is complex and largely remains to be investigated, but it is now widely accepted that environment, genetic background and immunity all contribute to the development of autoimmunity.

The ability of the immune system to avoid activation toward self-antigens is called tolerance. “Central” tolerance in the thymus and bone marrow plays a key role in shaping immune system homeostasis by inactivating or deleting autoreactive T and B lymphocytes. However, even under strict vigilance of “central” tolerance, small numbers of potentially self-reacting lymphocytes can still “leak out” into the periphery. This phenomenon does not necessarily lead to pathology because additional mechanisms of “peripheral” tolerance restrain the activation of these cells, including permanent inactivation of potentially autoreactive lymphocytes that recognize antigens in the absence of innate immune activation and inflammation (2). Any defect or failure in tolerance mechanisms can lead to breakdown of tolerance and to the development of autoimmunity (3). For example, some autoimmune diseases, such as the so called “interferonopathies,” are triggered by the recognition of self or foreign molecules by innate sensors (4, 5) which, in turn, trigger inflammation and engagement of previously quiescent autoreactive T and B cells (3).

Cytokines are crucial immune mediators that activate and polarize the immune response to grant host defense and recovery of homeostasis. On the other hand, excessive or persistent cytokine production results in deregulated immune activation and plays a role in both the initiation and the amplification phases of immunopathologies (6, 7). The key role of deregulated cytokine production in autoimmunity represents the rationale for therapeutic cytokine targeting with biologicals, an approach that has led to major successes in the treatment of diseases such as rheumatoid arthritis (RA) and psoriasis (8).

MicroRNAs (miRNAs) are a large family of short, non-coding, single stranded RNAs that regulate the expression of one third of human genes (9). As such, they play crucial roles in most physiological and pathological processes, including cell growth and differentiation, metabolism, immunity, cancer, and autoimmune disorders (10–12). Within the cell cytoplasm, miRNAs regulate gene expression post-transcriptionally by binding to complementary sequences in the coding, 5′- or 3′-untranslated region (UTR) of target mRNAs that is either silenced or degraded (9). In addition, miRNAs are now known to master cell-to-cell signaling via the association with extracellular vesicles that protect them from degradation and allow efficient entry into neighboring cells, where they regulate the expression of target mRNAs (13, 14). Interestingly, extracellular miRNAs were also shown to exert cell-to-cell regulation via a non-conventional mechanism consisting on the interaction with innate immune RNA sensors, such as Toll-like receptors 7 and 8 (TLR7 and TLR8) (15–17). Because of their tremendous potential as physiological and pathological regulators, miRNAs are in the limelight as promising future biopharmaceuticals (18).

In this review, we will summarize the literature describing miRNAs that influence the pathogenesis and course of autoimmune diseases by deregulating key pathogenic cytokines. In addition to shedding pathogenetic insights, our work may contribute to the identification of attractive candidate targets for the development of miRNA-based next generation drugs for immune-mediated pathologies.

## Search Methods

We searched the related articles indexed in PubMed database from inception to August 2018 using the following search details: (“micrornas”[MeSH Terms] OR “micrornas”[All Fields] OR “mirna”[All Fields]) AND (“cytokines”[MeSH Terms] OR “cytokines”[All Fields] OR “cytokine”[All Fields]) AND (“disease name”[MeSH Terms] OR “disease name”[All Fields]). We restricted our search to the best characterized autoimmune diseases, namely RA, systemic lupus erythematosus (SLE), psoriasis, Sjogren's syndrome (SS), type 1 diabetes, and multiple sclerosis (MS). Search results were screened for the source of analyzed miRNAs and cytokines. Works performed in cell lines stimulated to reproduce pathological tissue conditions were deliberately excluded. Original research papers clearly referring to basal miRNA and cytokine levels in pathology, either in the circulation/tissues or in cells from patients and murine models were selected to be discussed in paragraph 4 and summarized in Table 1. Additional literature was added, concerning cytokine biology and modulation in autoimmune diseases and miRNA biology, function and candidate therapeutic targets/tools.

**Table 1:** miRNAs involved in cytokine modulation in autoimmune diseases.

**Cytokine**	**miRNAs**	**Disease**	**Expression in disease**	**Source**	**Target**	**Effect on cytokine**	**References (PMID)**	**Mechanism**
TNF-α	GU-rich miRNAs	SLE	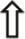	Plasma exosomes	TLR7	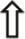	29769437	Direct effect (TLR activation)
		RA	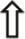	Synovial fluid of Macrophages	TLR7	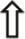	26662519	
	miR-10a	RA	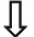	Synovium	TBX5	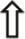	28782180	Indirect effect (activators)	
	miR-23b	RA-SLE-MS	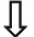	Synovia, renal biopsies, spinal cords	TAB3, TAB2, IKK-α	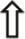	22660635	
	miR-155	RA	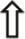	PBMC, monocytes, macrophages, synovial fluid	SOCS1; SHIP-1	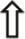	24351865; 27411480; 21690378	Indirect effect (repressors)
	miR-522	RA	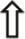	RASFs	SOCS3	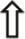	29394098	
	let-7a/e	SLE	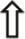	Kidney	TNFAIP3	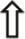	26110642	
	miR-21	PSO	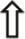	Lesional skin	TIMP3	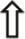	24574341	
	miR-106b	RA	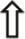	Ankle tissues from CIA mice	N.A.	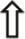	28957555	N.A.
	miR-146a	RA	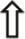	PBMC	N.A.	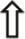	21810022	
	miR-155, miR132, miR-26a	MS	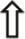	PBMC	N.A.	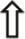	27310932	
	miR-125b	RA	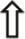	Serum, synovial tissues	N.A.	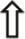	28738524	
IL-1β	miR-10a	RA	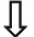	Synovium	TBX5	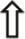	28782180	Indirect effect (activators)
	miR-23b	RA-SLE-MS	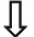	Synovia, renal biopsies, spinal cords	TAB3, TAB2, IKK-α	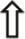	22660635	
	miR-155	RA	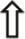	PBMC, monocytes, macrophages	SOCS1; SHIP-1	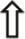	24351865; 21690378	Indirect effect (repressors)	
	miR-522	RA	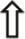	RASFs	SOCS3	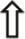	29394098	
	miR-31	PSO	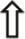	Lesional skin	STK40	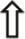	23233723	
	miR-448	MS	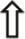	PBMC, cerebrospinal fluid (CSF)	PTPN2	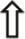	28342869	
	miR-106b	RA	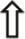	Ankle tissues from CIA mice	N.A	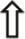	28957555	N.A.
	miR-125b	RA	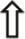	Serum, synovial tissues	N.A.	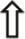	28738524	
IL-6	miR-410	SLE	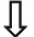	Kidney (SLE mouse model)	IL-6	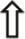	27028192	Direct effect
	GU rich miRNAs	SLE	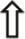	Plasma exosomes	TLR7	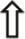	29769437	Direct effect (TLR activation)
		RA	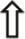	Synovial fluid of Macrophages	TLR7	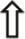	26662519	
	miR-10a	RA	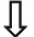	Synovium	TBX5	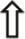	28782180	Indirect effect (activators)
	miR-140	RA	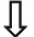	Synovial tissue and RASFs	TLR4	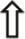	28987944	
	miR-22	RA	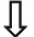	Synovial tissue	Cyr61	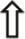	24449575	
	miR-23b	RA-SLE-MS	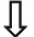	Synovia, renal biopsies, spinal cords	TAB3, TAB2, IKK-α	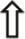	22660635	
	miR-155	RA	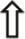	Monocytes, macrophages	SHIP-1	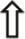	21690378	Indirect effect (repressors)
	miR-203	RA	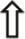	RASFs	NF-κB repressors and SOCS	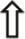	21279994	
	miR-106b	RA	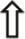	Ankle tissues from CIA mice	N.A.	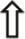	28957555	N.A.
IL-23	miR-21	PSO	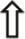	Lesional skin	TIMP-3	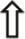	24574341	Indirect effect (repressors)
	miR-200a	PSO	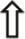	CD4+ T cells	N.A.	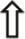	28738533	N.A.
IL-17	miR-340	PSO	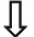	T cells (Imiquimod model)	IL-17A	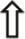	30012847	Direct effect
	miR-20b	MS	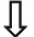	Th17 cells (EAE mice)	RORgt, STAT3	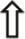	24842756	Indirect effect (activators)
	miR-30a	MS	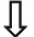	CD4+ T cells (EAE mice)	IRF4	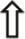	27581464	
	miR-210	PSO	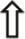	CD4+ T cells	FOXP3	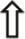	24316592	
	miR-451a	SLE	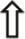	Spleen and thymus (mouse model)	IRF8	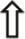	28120198	
	miR-326	MS	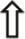	CD4+ T cells, EAE mice	Ets-1	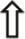	19838199	
	miR-26a	MS	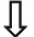	PBL of MS patients; brain of EAE mice	IL-6	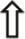	25362566	
	let-7e	MS	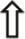	CD4+ T cells in EAE model	IL-10	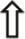	23079871	Indirect effect (repressors)
	miR-21	PSO	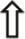	Lesional skin	TIMP3	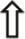	24574341	
	miR-448	MS	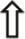	PBMC, CSF	PTPN2	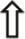	28342869	
	miR-155, miR132	MS	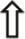	PBMC	N.A.	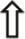	27310932	N.A.
	miR-200a	PSO-MS	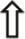	CD4+ T cells	N.A.	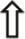	28738533; 25938517	
	miR-146a	PSO-RA	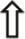	Lesional skin, PBMC, synovium	N.A.	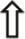	23018031; 20840794	
IFN-α	GU-rich miRNAs	SLE	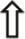	Plasma exosomes	TLR7	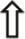	29769437	Direct effect (TLR activation)
	miR-146a	SLE	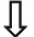	PBMC	IRF5, STAT1	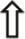	19333922	Indirect effect (activators)
	miR-302d	SLE	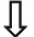	Monocytes	IRF9	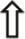	28318807	
	miR-155, miR-17 and miR-181b	SLE	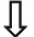	PBMC	N.A.	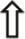	25775145	N.A.
IL-10	let-7e	MS	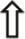	CD4+ T cells in EAE model	IL-10	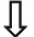	23079871	Direct effect
	miR-410	SLE	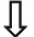	CD3+ T cells	STAT3	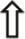	27351906	Indirect effect (activators)
	miR-223	RA	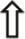	T cells	IGF-1R	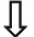	24816316	
	miR-210	PSO	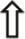	CD4+ T cells	FOXP3	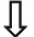	24316592	
	miR-21	SLE	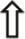	PBMC	PDCD4	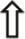	21602271	Indirect effect (repressors)

*SLE, Systemic Lupus Erythematosus; RA, Reumathoid Arthritis; RASF, RA Synovial Fluid; MS, Multiple Sclerosis; CSF, Cerebrospinal Fluid; PSO, Psoriasis; EAE, Experimental Autoimmune Encephalomyelitis; PBL, Peripheral Blood Lymphocyte; PBMC, Peripheral Blood Mononuclear Cell; CIA, Collagen-Induced Arthritis; N.A., Not Addressed*.

## Mechanisms of Cytokine Targeting By miRNAs

**Figure 1:**
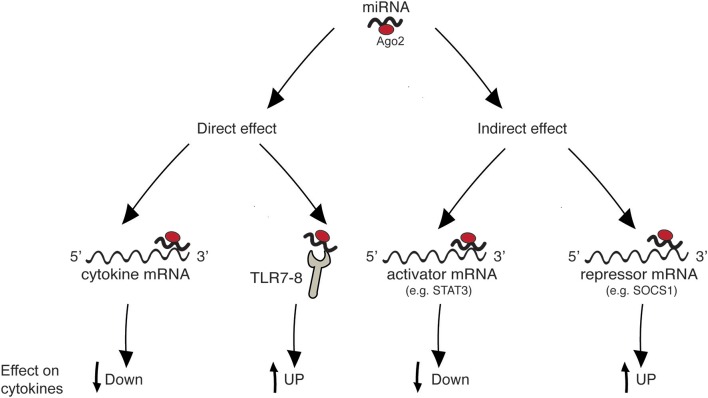
Mechanisms of cytokine regulation by miRNAs. “Direct” regulation comprises targeting of cytokine mRNA and triggering of innate immune receptors leading to cytokine production. “Indirect” regulation comprises targeting of molecules that act as inducers or inhibitors of a given cytokine.

Figure 1 summarizes the four main mechanisms through which miRNAs regulate cytokine levels. Regulation can be either “direct” on the target cytokine, or “indirect,” meaning that one given miRNA post-transcriptionally regulates the expression of a protein that in turn influences the level of the cytokine. “Direct” regulation comprises both the targeting of cytokine mRNA, reflecting in decreased cytokine levels, and the stimulation of TLR7/8, reflecting in cytokine increase. In “indirect” regulation, if one miRNA targets a cytokine activator, the cytokine level is expected to be decreased. By contrast, if a repressor is targeted the cytokine level increases. However, as discussed in specific paragraphs and summarized in Table 1, the neat result in terms of cytokine production also depends on the level of the analyzed miRNA in the specific pathology (increased or decreased in respect to healthy individuals). Please note that, in the present review, the terms “repressor” and “activator” are intended in their wider meaning and one single protein may be considered repressor or activator depending on the cytokine under consideration [e.g., FOXP3 is considered “activator” for IL-17 production and “repressor” of IL-10, based on its role of Th17-promoting transcription factor (19), see Table 1].

## miRNA-Mediated Cytokine Targeting in Autoimmune Diseases

### Primary Pro-Inflammatory Cytokines (TNF-α, IL-1β, IL-6)

Primary pro-inflammatory cytokines are increased in RA patients and play a vital role in the pathogenesis of this disease, characterized by chronic inflammation of the synovial tissue, joint dysfunction, and tissue damage in the joints. Collectively, these cytokines facilitate the recruitment of leukocytes into the joints to maintain chronic inflammation, induce the proliferation of synovial fibroblasts that leads to pannus formation and contribute to angiogenesis and cartilage and bone destruction in the course of arthritis (7, 8). However, pro-inflammatory cytokines also display non-overlapping pathogenic functions that are not fully understood in autoimmunity. Indeed, while TNF-α and IL-β inhibition turned out to be effective approaches in the treatment of RA and of other chronic arthritis, the therapeutic effect of IL-1 inhibition proved unexpectedly modest (8, 20).

miRNAs were described to play a role in the pathogenic increase of pro-inflammatory cytokines in RA, but also in SLE, psoriasis and MS. A few reports describe miRNA-mediated “direct” regulation, while many more demonstrate “indirect” modulation of pro-inflammatory cytokine production, either via the targeting of activators or of repressors (Figure 1 and Table 1).

Direct mRNA targeting was shown in kidneys of MRL/lpr SLE mouse model, where increased IL-6 levels depended on a decrease in miR-410, which targeted the 3′-UTR region of IL-6 mRNA (21).

Two groups reported a role for TLR7 stimulation in increased pro-inflammatory cytokine secretion in autoimmune conditions. Let-7b was markedly upregulated in synovial fluid of patients with RA and capable of inducing TNF-α and IL-6 production by macrophages via TLR7 ligation (22). Our own group recently demonstrated that TNF-α and IL-6 are produced by human primary plasmacytoid dendritic cells (pDCs) stimulated with exosomes isolated from plasma of SLE patients. This effect depends on the triggering of TLR7 by exosome-associated miRNAs (17). Both groups found that TLR7 triggering can be mediated by several miRNAs rich in guanosine and uridine. This is in line with recent structural studies demonstrating that TLR7 works as a dual sensor for guanosine and uridine-containing ssRNAs by associating with degradation products of RNA instead of recognizing specific RNA sequences [reviewed in (23)].

In the synovium of RA patients, the down-modulation of miR-10a promoted the expression of TBX5, a member of T-box transcription factor family. TBX5 is an important regulator of synovial fibroblast that in turn increased the expression of TNF-α, IL-6, and IL-1β (24).

miR-23b was found down-regulated in human lesions and in murine models of SLE and RA, as well as in a model of MS. This suppression depended on IL-17 and contributed to autoimmune inflammation by promoting the expression of pro-inflammatory cytokines. Indeed, miR-23b suppresses NF-κB activation and inflammatory cytokine expression by targeting TGF-β-activated kinase 1/MAP3K7 binding protein 2 (TAB2), TAB3 and inhibitor of nuclear factor κ-B kinase subunit α (IKK-α). As expected, these second messengers that are essential in the pathway leading to inflammatory NF-κB activation were upregulated both in RA patients and in murine models (25).

miR-155 was increased in peripheral blood mononuclear cells (PBMCs) (26), peripheral blood monocytes (27) and synovial macrophages and monocytes (28) isolated from RA patients as compared with healthy controls. Increased miR-155 could increase the expression of pro-inflammatory cytokines by targeting Suppressor of cytokine signaling 1 (SOCS1) (26) and Src homology 2-containing inositol phosphatase-1 (SHIP-1) (28) in the respective cell type. Similarly, miR-522 and miR203, which are up-regulated in synovial fibroblasts of RA patients, respectively, increased the expression of TNF-α and IL-1β via targeting SOCS3 (29) and of IL-6 by targeting inhibitors of the NF-κB pathway, although these could not be further identified (30).

IL-6 production was also stimulated by two other miRNAs, miR-140 and miR-22, both down-regulated in synovial tissue samples from RA patients. In the case of miR-140, IL-6 upregulation was induced by a significant increase of TLR4, its direct target (31). Indeed, it is well-established that the slightest increase in the expression of TLRs may translate in overt autoimmune phenotypes [reviewed in (32)]. miR-22 expression was found to negatively correlate with that of Cyr61, a secreted extracellular matrix protein that promotes fibroblast-like synoviocyte proliferation. This increased IL-6 production and consequent Th17 differentiation (33).

Let-7 deregulation was reported to influence SLE pathogenesis. In particular, let-7a and let-7e were up-regulated in kidney biopsies of SLE patients independent of lupus nephritis and increased the production of TNF-α by suppressing TNF-α Induced Protein 3 (TNFAIP3), an ubiquitin-editing enzyme that negatively regulates the activation of NF-κB (34).

miR-21 and miR31 were involved in increased expression of pro-inflammatory cytokines in psoriasis. Increased miR-21 levels in epidermal lesions of psoriatic patients correlates with increased expression of TNF-α, because of reduced expression of epidermal Tissue Inhibitor of Metalloproteinase 3 (TIMP3) and consequent activation of TNF-α Converting Enzyme (TACE), responsible for the shedding of the functional ectodomain of TNF-α from cell membranes (35). miR-31, markedly over-expressed in psoriatic keratinocytes, was responsible for IL-1β over-expression, as demonstrated by the block obtained with an anti-miR31. The authors found that increased miR31 suppressed Serine/Threonine Kinase 40 (STK40), a suppressor of NF-κB activation (36).

miR-448 is significantly increased in both PBMCs and cerebrospinal fluid of patients with MS and enhances the production of pro-inflammatory cytokines, including IL-1β and IL-17, through targeting protein tyrosine phosphatase non-receptor type 2 (PTPN2) thus promoting Th17 differentiation (37).

In addition to the evidence discussed above, the levels of additional miRNA were found to correlate with pro-inflammatory cytokine expression, although the mechanisms remained not addressed. In collagen induced arthritis, mice displayed increased expression of miR-106b, an important miRNA involved in bone remodeling (38). miR-106 inhibition led to decreases arthritis severity and reduced levels of serum pro-inflammatory cytokines (39). In PBMCs of MS patients, the upregulation of miR-155, miR-132, and miR-26a associated to increased expression of TNF-α and IL-17 (40). Finally, in patients with RA the expression of miR-146a and 125b was increased as compared to healthy controls and positively correlated with levels of pro-inflammatory cytokines (41, 42).

All in all, these studies indicate that TNF-α, IL-1β, and IL-6 are relevant targets of miRNAs that are deregulated in autoimmune diseases. Because these cytokines share most of the inducing stimuli and pathways, miRNAs acting via indirect mechanism are often found to regulate all of them. Thus, miRNAs could represent relevant deregulators of pro-inflammatory cytokines and, as such, interesting therapeutic targets for controlling their aberrant production in autoimmune diseases. However, our survey also shows that, at present, it is not possible to identify one or a small group of miRNAs representing the miRNA signature of the disease, i.e., the miRNAs mainly responsible for pro-inflammatory cytokine deregulation and possible therapeutic candidate/s. Indeed, single reports investigate different aspects or cell types within the different diseases making it difficult to gain an integrated view of cytokine deregulation by miRNAs.

### The IL-23/IL-17 Axis

IL-23 is a crucial player in T-cell-mediated responses and a key promoter of immune-mediated pathological conditions. With the requisite assistance of other cytokines such as IL-6 and TGF-β, IL-23 masters the polarization of naïve CD4^+^ T cells into Th17 effector cells (43). Many other innate immune cells characterized by the expression of the transcription factor RORγt and γδ T cells are also responsive to IL-23 (44). Collectively, these cells are responsible for the production of inflammatory cytokines including IL-17, IL-22, and TNF-α, inciting local tissue inflammation and immune-mediated inflammatory conditions. Aberrant IL-17 production has been identified in many autoimmune diseases including psoriasis, inflammatory bowel disease, RA, and MS (45). Consistently, IL23/IL-17 axis blockade is a successful therapy for psoriasis and psoriatic arthritis.

A direct regulatory effect of this axis was described for miR-340, which controls the expression of endogenous IL-17A by specifically binding to its 3′ UTR. miR-340 was decreased in T cells from the Imiquimod psoriasis mouse model, thus increasing the release of IL-17A. Furthermore, treatment with miR-340 alleviated the clinical severity of Imiquimod-induced psoriasis (46).

Many other miRNAs were found to regulate the IL-23/IL-17 axis in autoimmune diseases by indirect mechanisms.

In experimental autoimmune encephalomyelitis (EAE) miR-20b, miR-30a, and miR-26a were reduced. Decreased miR-30a and miR-26a was confirmed also in peripheral blood CD4^+^ T cells of MS patients (47, 48). miR-20b was shown to suppress Th17 differentiation *in vitro* and *in vivo* by targeting RORγt and STAT3, thus acting as a negative regulator of EAE (49). Similarly, over-expression of miR-30a inhibited Th17 differentiation and prevented the full development of EAE, whereas interference of miR-30a promoted Th17 differentiation. miR-30a was shown to reduce IRF4 expression by specifically binding its 3′-UTR (47). miR-26a was shown to be a IL-6-associated miRNA and therefore an indirect regulator of the Th17/Treg cells balance, which inhibition substantially aggravated EAE severity (48). miR-326 and Let-7e were significantly up-regulated EAE. miR-326 expression also correlated with disease severity in MS patients. It was shown to promote the generation of Th17 cells by targeting Ets-1, a negative regulator of Th17 cell differentiation (50). Let-7e indirectly enhanced IL-17 production by targeting the 3′UTR of IL-10 mRNA (51).

CD4^+^T cells from patients with psoriasis vulgaris showed miR-200a and miR-210 over-expression. miR-200a expression positively correlated with that of RORγT, IL-17, IL-23 (52, 53). miR-210 deregulation led to decreased IL-10 and increased IL-17 production, thus impairing the immunosuppressive functions of Treg cells, via the inhibition of FOXP3 expression (54).

In lesional skin from psoriatic patients miR-21 was up-regulated. Anti-miR-21 treatment of mice receiving patient-derived xenotransplants resulted in IL-17 and IL-23 down-regulation (35). Similarly, miR-146a was up-regulated in lesional skin and PBMCs of psoriatic patients (55), but also in RA synovium (56), and positively correlated with IL-17 expression and disease severity (55, 56).

miR-451a expression was increased in spleen and thymus of a SLE mouse and its blockade decreases serum level of IL-17. *In vitro* and *in vivo* studies identified IRF8 as a target of miR-451a (57).

### Type I IFNs

Type I IFNs are a family of cytokines produced by innate immune cells (pDCs in particular) and by tissue cells upon sensing of viral nucleic acids via RIG-Like Receptors (RLRs) and TLRs. By binding to a common, ubiquitously expressed receptor, these cytokines induce viral resistance in tissues and exert important immunostimulatory functions (58). Increased levels of type I IFNs are the hallmark and a pathogenic mechanism of a class of autoimmune diseases known as “interferonopathies” comprising SLE, psoriasis, SS, and others (5, 6, 59). Indeed, several inhibitors of type I IFN are currently under clinical trial for the treatment of SLE and psoriasis (6).

A direct regulation of type I IFN production by miRNAs was described by our own group. Indeed, together with pro-inflammatory cytokines, we found that exosome-associated miRNAs from the plasma of inactive SLE patients induced also the release of type I IFNs by human primary pDCs via TLR7 triggering (17).

A decreased expression of several miRNAs was implicated in the over-expression of type I IFNs in SLE patients. Under-expression of miR-146a, a negative regulator of innate immunity, in both active and inactive patients negatively correlated with clinical disease activity and with IFN scores. However, in active patients the levels were significantly lower than in inactive individuals. In healthy PBMCs, inhibition of endogenous miR-146a increased the induction of type I IFNs, while over-expression repressed type I IFN production by targeting IRF5 and STAT1. Importantly, introduction of miR-146a into the patients' PBMCs alleviated the coordinate activation of the type I IFN pathway (60).

miR-302d is an estrogen-regulated miRNA that was found decreased in SLE monocytes, where it inversely correlated with the IFN-dependent genes MX1 and OAS1. It also inversely correlated with the levels of its predicted target, IRF9, a critical component of the transcriptional complex that regulates expression of genes induced by type I IFNs. Furthermore, significantly reduced miR-302d levels and increased IRF9 levels were identified in SLE patients with active disease as compared to inactive individuals (61).

Another study found a strong inverse correlation between type I IFNs expression and the levels of miR-155, miR-17, and miR-181b in PBMCs of active SLE patients, but the molecular mechanism was not elucidated (62).

### IL-10

IL-10 is a pleiotropic cytokine produced by multiple cell types including innate immune cells, B cells, Th1, and Th2 cells, CD4^+^CD25^+^FOXP3^+^ Treg cells, and keratinocytes (63). It exerts anti-inflammatory and immunomodulatory effects mainly acting on innate myeloid cells. Indeed, IL-10 directly inhibits the production of primary pro-inflammatory cytokines, thus representing a key anti-inflammatory mediator. In addition, it indirectly inhibits the activation of adaptive immunity also by blocking the production of IL-12 and the expression of MHC and costimulatory molecules. Ultimately, IL-10 is thought to play a crucial role in terminating excessive T-cell responses to prevent chronic inflammation and tissue damage, especially at the mucosal level (64), as demonstrated by the observation that IL-10-deficient mice develop spontaneous enterocolitis and other Crohn's disease-like symptoms as well as exaggerated asthmatic and allergic responses (65).

let-7e is significantly up-regulated in EAE and directly decreases IL-10 production by targeting its 3′UTR (51).

In activated T cells from RA patients, increased levels of miR-223 were implicated in decreased production of IL-10. This effect depended on IGF-1R targeting by miR-223. Indeed, IL-10 secretion was shown to depend on IGF in these cells (66).

CD4^+^ T cells from patients with psoriasis vulgaris showed miR-210 over-expression. This study showed that miR-210 inhibits FOXP3 expression, thus impairing the immunosuppressive functions of Treg cells and decreasing the levels of IL-10 (54).

Elevated IL-10 levels were shown to correlate with disease activity in SLE (67) miR-410. was down-regulated in CD3^+^T cells of SLE patients as compared to healthy controls and was shown to target the 3′ UTR of STAT3 mRNA. This would result in increased STAT3 levels, which is a positive regulator of IL-10 production in CD3^+^T cells (68). Also, miR-21 upregulation strongly correlated with SLE disease activity. Its silencing decreased IL-10 production by T cells. Investigation of putative gene-targets showed PDCD4 (a selective protein translation inhibitor) to be effectively suppressed by miR-21. Accordingly, PDCD4 expression was confirmed to be decreased in active SLE (69).

## miRNAs AS Future Therapeutics

Cytokine targeting with monoclonal antibodies or recombinant peptides is nowadays a powerful therapeutic option for autoimmune diseases that is dramatically improving patient outcomes (70). However, it does not work for everyone: in the case of RA, for example, improvement is usually seen in about two thirds of patients and it remains impossible to predict which patients will benefit of the treatment. In addition, high costs and lack of oral absorption have often represented major barriers for the success of biologicals (71).

The discovery of miRNAs as important regulatory agents for gene expression and their widespread deregulation in several pathological settings boosted the idea to exploit them as therapeutic targets and tools [reviewed in (18, 72–74)]. Available literature confirms that cytokines are relevant targets of miRNAs that are deregulated in autoimmune diseases. Thus, miRNAs could represent interesting therapeutic targets for controlling aberrant cytokine production involved in the onset and amplification of autoimmunity. However, at present, it is not possible to identify signature miRNAs, i.e., the miRNAs mainly responsible for cytokine deregulation in specific autoimmune diseases to be addressed as therapeutic candidate/s.

miRNAs possess unique characteristics that render them very attractive in terms of drug development (72). First, they are small, with known sequences and are often conserved among species. Second, it is possible both to supplement downregulated miRNAs by using synthetic oligonucleotides and to block the effects of increased miRNAs through artificial antagonists, either oligonucleotides or small molecules. In this regard, miRNA-based therapies can also take advantage from decades of research on other therapeutic oligonucleotides. Third, the ease of administration through local or parenteral injection routes and sufficient uptake in tissues gives miRNA therapeutics an extra edge. Last, but not least, one single miRNA can regulate different targets and potentially influence entire cellular pathways or processes. However, our current lack of a full understanding of miRNA biology and of the intricate network of interactions between miRNAs and the human genome, transcriptome and proteome restrains the translation of miRNA-based therapy into the clinical use. Also, as above anticipated, the identification and validation of signature miRNAs has yet to come for most diseases. In addition, a number of specific challenges associated with miRNA targeting still need to be faced, such as predicting possible off-target effects and toxicity, improving miRNA stability and optimizing the delivery systems.

In the last 5 years, a number of miRNA-based therapeutic tools entered in clinical trials, mainly for cancer management (73–75). Thus, an increasing amount of preclinical and clinical data for miRNA replacements and antagonists is expected to become soon available. This, together with progresses in characterizing disease-signature miRNAs, will determine the therapeutic future of this potentially powerful technology.

## Concluding Remarks

Altered miRNA levels are observed in most autoimmune diseases and are recognized to influence autoimmunity through different mechanisms, among which deregulation of pathogenic cytokines may be of crucial importance. Literature describing novel deregulated miRNAs and putative targets is tumultuously growing. Although much work has still to be performed to gain an integrated overview of the relevant miRNAs and molecular mechanisms of cytokine modulation in specific autoimmune diseases, these studies will hopefully lead to the identification of disease-specific signature miRNAs. These, in turn, will represent interesting candidates for next generation drugs aimed at controlling the production of pathogenic cytokines in autoimmune conditions.

## Author Contributions

DB conceived the article and wrote the manuscript. VS substantially contributed to draft writing and prepared the table. VG contributed to draft writing and table editing and provided artwork. LT contributed to draft writing. SS conceived the work and contributed critical revision of the manuscript. All authors approved the final version of the manuscript.

### Conflict of Interest Statement

The authors declare that the research was conducted in the absence of any commercial or financial relationships that could be construed as a potential conflict of interest.
